# *N*-(2-Nitro­phen­yl)furan-2-carboxamide

**DOI:** 10.1107/S1600536813026202

**Published:** 2013-09-28

**Authors:** Rodolfo Moreno-Fuquen, Alexis Azcárate, Alan R. Kennedy, Denise Gilmour, Regina H. De Almeida Santos

**Affiliations:** a Departamento de Química – Facultad de Ciencias, Universidad del Valle, Apartado 25360, Santiago de Cali, Colombia; b WestCHEM, Department of Pure and Applied Chemistry, University of Strathclyde, 295 Cathedral Street, Glasgow G1 1XL, Scotland; c Instituto de Química de São Carlos, IFSC, Universidade de São Paulo, USP, São Carlos, SP, Brazil

## Abstract

In the title furan­carboxamide derivative, C_11_H_8_N_2_O_4_, the benzene and furan rings are rotated from the mean plane of the central fragment by 2.68 (5) and 7.03 (4)°, respectively. The nitro group forms a dihedral angle of 10.15 (5)° with the adjacent benzene ring. In the crystal, mol­ecules are linked by weak C—H⋯O inter­actions, forming helical chains running along [010].

## Related literature

For similar furan­carboxamide compounds, see: Pavlović *et al.* (2004) ▶ and for similar 2-nitro­phenyl­amino compounds, see: Glidewell *et al.* (2004 ▶). For hydrogen-bonding information, see: Nardelli (1995 ▶). For hydrogen-bond motifs, see: Etter *et al.* (1990 ▶). For a description of the Cambridge Structural Database, see: Allen (2002 ▶).
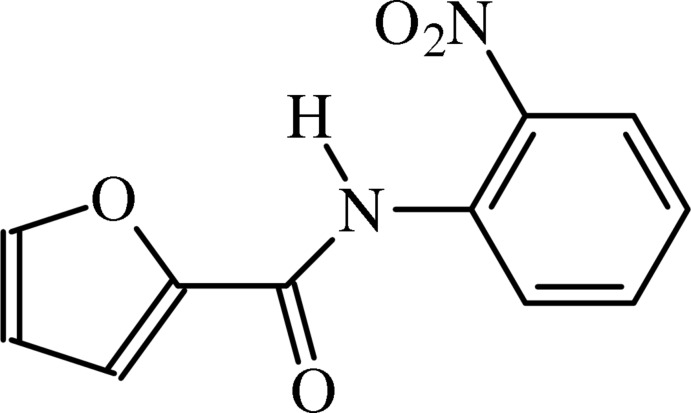


## Experimental

### 

#### Crystal data

C_11_H_8_N_2_O_4_
*M* *_r_* = 232.19
Monoclinic, 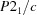

*a* = 7.0380 (5) Å
*b* = 12.8072 (9) Å
*c* = 11.3701 (9) Å
β = 97.819 (6)°
*V* = 1015.34 (13) Å^3^
*Z* = 4
Mo *K*α radiation
μ = 0.12 mm^−1^
*T* = 123 K
0.35 × 0.33 × 0.25 mm

#### Data collection

Oxford Diffraction Xcalibur E diffractometer
4090 measured reflections
2649 independent reflections
1859 reflections with *I* > 2σ(*I*)
*R*_int_ = 0.016

#### Refinement

*R*[*F* ^2^ > 2σ(*F* ^2^)] = 0.039
*wR*(*F* ^2^) = 0.099
*S* = 1.04
2649 reflections
158 parameters
H atoms treated by a mixture of independent and constrained refinement
Δρ_max_ = 0.21 e Å^−3^
Δρ_min_ = −0.26 e Å^−3^

### 

Data collection: *CrysAlis PRO* (Oxford Diffraction, 2010 ▶); cell refinement: *CrysAlis PRO*; data reduction: *CrysAlis PRO*; program(s) used to solve structure: *SHELXS97* (Sheldrick, 2008 ▶); program(s) used to refine structure: *SHELXL97* (Sheldrick, 2008 ▶); molecular graphics: *ORTEP-3 for Windows* (Farrugia, 2012 ▶) and *Mercury* (Macrae *et al.*, 2006 ▶); software used to prepare material for publication: *WinGX* (Farrugia, 2012 ▶).

## Supplementary Material

Crystal structure: contains datablock(s) I, global. DOI: 10.1107/S1600536813026202/gg2129sup1.cif

Structure factors: contains datablock(s) I. DOI: 10.1107/S1600536813026202/gg2129Isup2.hkl

Supplementary material file. DOI: 10.1107/S1600536813026202/gg2129Isup3.cml

Additional supplementary materials:  crystallographic information; 3D view; checkCIF report

## Figures and Tables

**Table 1:** Hydrogen-bond geometry (Å, °)

*D*—H⋯*A*	*D*—H	H⋯*A*	*D*⋯*A*	*D*—H⋯*A*
C2—H2⋯O2^i^	0.95	2.55	3.3857 (18)	146

Symmetry code: (i) 
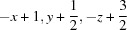
.

**Fig. 1.**
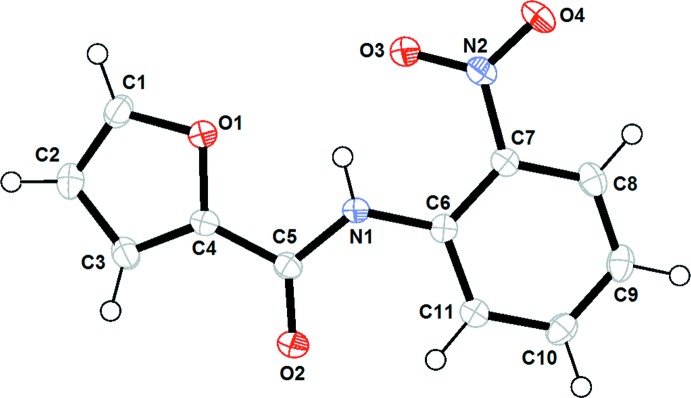
Molecular conformation and atom numbering scheme for the title compound with displacement ellipsoids drawn at the 50% probability level. H atoms are shown as spheres of arbitrary radius.

**Fig. 2.**
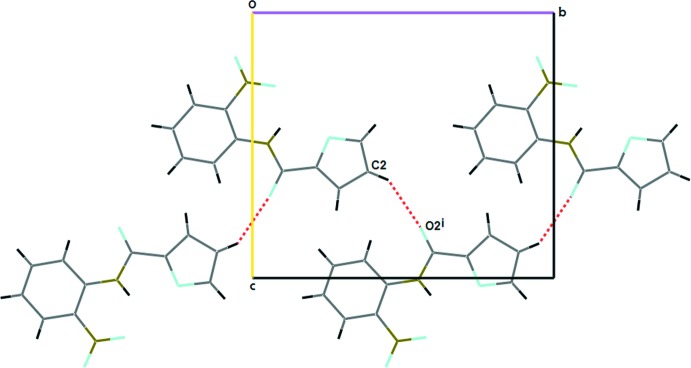
Part of the crystal structure of (I), showing the formation of helical chains which running along [010]. Symmetry code: (i) -x+1,+y+1/2,-z+3/2.

## supplementary crystallographic information

### 1. Comment

In the present work, the structure of N-(2-nitrophenyl)-2-furancarboxamide (I) has been determined as a part of a study undertaken in our research group on 2-nitrophenyl substituted carboxamides. Similar structures are known from the literature: 2-furancarboxanilide (2FCCA) [Pavlović *et al.*, 2004] and ortho nitrophenylaminocarbonyl benzoic acid (2NPCB) [Glidewell *et al.*, 2004] and they serve as a comparison to the values of the title system (I). The molecular structure of (I) is shown in Fig. 1. The central C4-C5(O2)-N1-C6 fragment of the molecule is essentially planar with a trans amide conformation. This behavior agrees with the behavior presented by 2NPCB system. The phenyl and furan rings are rotated from the mean plane of the central fragment by 2.68 (5)° and 7.03 (4)° respectively. The dihedral angle between the phenyl and furan rings is 9.71 (5)°. The nitro group forms a dihedral angle with the adjacent benzene ring of 10.15 (5)°. The formation of relatively strong intramolecular bonds between the central fragment and the furan ring, in some similar systems, can preserve the planarity of the 2-furancarboxamide moiety [Pavlović *et al.*, 2004]. However, the planarity of the 2-furancarboxamide moiety is not preserved in the title compound. Relatively strong intramolecular interactions between the central fragment and nitrophenyl ring are observed. Indeed, the intramolecular interaction with the nitro group [N1···O3, 2.615 (1) Å and 135 (1)°] forces the central fragment to rotate, relative to the furan ring. The C1-C2 and C3-C4 within the furan ring, are within the expected range [1.341 Å; Allen *et al.*, 2002]. Other bond distances in both furan and the phenyl rings are consistent with expected values (Allen *et al.*, 2002). In the crystal, the molecules of (I) are linked by weak C2-H2···O2 interactions, forming one-dimensional helical chains running along [010], as shown in Fig. 2. The atom C2 of the furan ring at (x,y,z) acts as a hydrogen-bond donor to carbonyl atom O2 at (-x+1,+y+1/2,-z+3/2) (see Nardelli, 1995), forming a pattern specified as C(6) (Etter, 1990).

### 2. Experimental

The reagents and solvents for the synthesis were obtained from the Aldrich Chemical Co., and were used without additional purification. The title molecule was synthesized using equimolar quantities of furan-2-carbonyl chloride (0.202 g, 1.548 mmol) and 2-nitroaniline (0.144 g). The reagents were dissolved in 10 mL of acetonitrile and the solution was taken to reflux in constant stirring for 3 hours. Yellow crystals of good quality were obtained after leaving the solvent to evaporate. IR spectra were recorded on a FT—IR SHIMADZU IR-Affinity-1 spectrophotometer. m.p 388 (1) K. IR (KBr) 3310.91 cm^-1^ (amide N-H), 3127.48 cm^-1^ (aromatic C—H); 1679.05 cm^-1^ (amide C=O); 1594.47 cm^-1^, 1504.96 cm^-1^ (-NO_2_).

### 3. Refinement

Crystal data, data collection and structure refinement details are summarized in Table 1. All H-atoms were positioned at geometrically idealized positions with C—H distance of 0.95 Å and U_iso_(H) = 1.2 times U_eq_ of the C-atoms to which they were bonded. The coordinates of the H1N atom were refined.

### Crystal data

**Table:**

C_11_H_8_N_2_O_4_	*F*(000) = 480
*M**_r_* = 232.19	*D*_x_ = 1.519 Mg m^−^^3^
Monoclinic, *P*2_1_/*c*	Melting point: 388(1) K
Hall symbol: -P 2ybc	Mo *K*α radiation, λ = 0.71073 Å
*a* = 7.0380 (5) Å	Cell parameters from 4090 reflections
*b* = 12.8072 (9) Å	θ = 3.2–28.8°
*c* = 11.3701 (9) Å	µ = 0.12 mm^−^^1^
β = 97.819 (6)°	*T* = 123 K
*V* = 1015.34 (13) Å^3^	Block, colourless
*Z* = 4	0.35 × 0.33 × 0.25 mm

### Data collection

**Table:**

Oxford Diffraction Xcalibur E diffractometer	1859 reflections with *I* > 2σ(*I*)
Radiation source: fine-focus sealed tube	*R*_int_ = 0.016
Graphite monochromator	θ_max_ = 28.8°, θ_min_ = 3.2°
ω scans	*h* = −9→8
4090 measured reflections	*k* = −16→17
2649 independent reflections	*l* = −7→15

### Refinement

**Table:**

Refinement on *F*^2^	Primary atom site location: structure-invariant direct methods
Least-squares matrix: full	Secondary atom site location: difference Fourier map
*R*[*F*^2^ > 2σ(*F*^2^)] = 0.039	Hydrogen site location: inferred from neighbouring sites
*wR*(*F*^2^) = 0.099	H atoms treated by a mixture of independent and constrained refinement
*S* = 1.04	*w* = 1/[σ^2^(*F*_o_^2^) + (0.0395*P*)^2^ + 0.3116*P*] where *P* = (*F*_o_^2^ + 2*F*_c_^2^)/3
2649 reflections	(Δ/σ)_max_ < 0.001
158 parameters	Δρ_max_ = 0.21 e Å^−^^3^
0 restraints	Δρ_min_ = −0.26 e Å^−^^3^

### Special details

**Table:**

Geometry. All esds (except the esd in the dihedral angle between two l.s. planes) are estimated using the full covariance matrix. The cell esds are taken into account individually in the estimation of esds in distances, angles and torsion angles; correlations between esds in cell parameters are only used when they are defined by crystal symmetry. An approximate (isotropic) treatment of cell esds is used for estimating esds involving l.s. planes.
Refinement. Refinement of *F*^2^ against ALL reflections. The weighted *R*-factor *wR* and goodness of fit *S* are based on *F*^2^, conventional *R*-factors *R* are based on *F*, with *F* set to zero for negative *F*^2^. The threshold expression of *F*^2^ > σ(*F*^2^) is used only for calculating *R*-factors(gt) *etc*. and is not relevant to the choice of reflections for refinement. *R*-factors based on *F*^2^ are statistically about twice as large as those based on *F*, and *R*- factors based on ALL data will be even larger.

### Fractional atomic coordinates and isotropic or equivalent isotropic displacement parameters (Å^2^)

**Table:**

	*x*	*y*	*z*	*U*_iso_*/*U*_eq_	
O1	0.33408 (13)	0.25426 (7)	0.47056 (8)	0.0224 (2)	
O2	0.39374 (15)	0.05694 (8)	0.69240 (9)	0.0285 (3)	
O3	0.10004 (16)	0.07661 (8)	0.27664 (9)	0.0296 (3)	
O4	0.02689 (18)	−0.05720 (9)	0.16525 (9)	0.0374 (3)	
N1	0.27083 (16)	0.05097 (9)	0.49375 (10)	0.0188 (3)	
N2	0.08218 (17)	−0.01884 (10)	0.26244 (10)	0.0228 (3)	
C1	0.3632 (2)	0.35951 (11)	0.48419 (14)	0.0263 (3)	
H1	0.3458	0.4089	0.4213	0.032*	
C2	0.4197 (2)	0.38338 (12)	0.59859 (14)	0.0270 (3)	
H2	0.4482	0.4510	0.6306	0.032*	
C3	0.4287 (2)	0.28777 (12)	0.66254 (13)	0.0244 (3)	
H3	0.4647	0.2788	0.7455	0.029*	
C4	0.37595 (18)	0.21213 (11)	0.58195 (12)	0.0190 (3)	
C5	0.34973 (18)	0.09956 (11)	0.59629 (11)	0.0190 (3)	
C6	0.21685 (18)	−0.05325 (11)	0.47586 (12)	0.0177 (3)	
C7	0.12627 (18)	−0.08875 (11)	0.36456 (12)	0.0191 (3)	
C8	0.07181 (19)	−0.19269 (12)	0.34607 (13)	0.0231 (3)	
H8	0.0103	−0.2145	0.2705	0.028*	
C9	0.1070 (2)	−0.26394 (11)	0.43720 (13)	0.0252 (3)	
H9	0.0696	−0.3348	0.4252	0.030*	
C10	0.1977 (2)	−0.23083 (11)	0.54624 (13)	0.0239 (3)	
H10	0.2235	−0.2800	0.6089	0.029*	
C11	0.25161 (19)	−0.12788 (11)	0.56631 (12)	0.0209 (3)	
H11	0.3131	−0.1075	0.6424	0.025*	
H1N	0.239 (2)	0.0920 (14)	0.4345 (16)	0.036 (5)*	

### Atomic displacement parameters (Å^2^)

**Table:**

	*U*^11^	*U*^22^	*U*^33^	*U*^12^	*U*^13^	*U*^23^
O1	0.0275 (5)	0.0196 (5)	0.0194 (5)	−0.0012 (4)	0.0004 (4)	0.0016 (4)
O2	0.0413 (6)	0.0249 (6)	0.0174 (5)	−0.0017 (5)	−0.0024 (4)	0.0014 (4)
O3	0.0428 (7)	0.0219 (6)	0.0223 (6)	0.0006 (5)	−0.0018 (4)	0.0005 (4)
O4	0.0554 (8)	0.0350 (7)	0.0188 (6)	−0.0021 (6)	−0.0061 (5)	−0.0061 (5)
N1	0.0222 (6)	0.0177 (6)	0.0161 (6)	0.0003 (5)	0.0007 (4)	0.0012 (5)
N2	0.0235 (6)	0.0260 (7)	0.0185 (6)	0.0012 (5)	0.0017 (5)	−0.0020 (5)
C1	0.0279 (8)	0.0188 (7)	0.0318 (8)	−0.0014 (6)	0.0021 (6)	0.0032 (6)
C2	0.0291 (8)	0.0211 (7)	0.0312 (8)	−0.0040 (6)	0.0048 (6)	−0.0044 (6)
C3	0.0249 (7)	0.0257 (8)	0.0225 (7)	−0.0044 (6)	0.0033 (5)	−0.0042 (6)
C4	0.0174 (6)	0.0225 (7)	0.0171 (7)	0.0002 (5)	0.0022 (5)	0.0008 (5)
C5	0.0178 (6)	0.0217 (7)	0.0176 (7)	0.0000 (6)	0.0027 (5)	−0.0007 (6)
C6	0.0144 (6)	0.0193 (7)	0.0200 (7)	0.0006 (5)	0.0047 (5)	−0.0007 (5)
C7	0.0170 (6)	0.0216 (7)	0.0191 (7)	0.0011 (5)	0.0039 (5)	−0.0011 (6)
C8	0.0196 (7)	0.0268 (8)	0.0237 (7)	−0.0025 (6)	0.0060 (5)	−0.0070 (6)
C9	0.0249 (7)	0.0199 (7)	0.0322 (8)	−0.0046 (6)	0.0096 (6)	−0.0034 (6)
C10	0.0245 (7)	0.0211 (7)	0.0274 (8)	0.0000 (6)	0.0079 (6)	0.0042 (6)
C11	0.0203 (7)	0.0227 (7)	0.0196 (7)	−0.0001 (6)	0.0022 (5)	−0.0004 (6)

### Geometric parameters (Å, º)

**Table:**

O1—C1	1.3690 (17)	C3—C4	1.3503 (19)
O1—C4	1.3713 (16)	C3—H3	0.9500
O2—C5	1.2231 (16)	C4—C5	1.4653 (19)
O3—N2	1.2371 (16)	C6—C11	1.4009 (19)
O4—N2	1.2235 (15)	C6—C7	1.4125 (18)
N1—C5	1.3706 (17)	C7—C8	1.393 (2)
N1—C6	1.3952 (17)	C8—C9	1.378 (2)
N1—H1N	0.860 (18)	C8—H8	0.9500
N2—C7	1.4656 (18)	C9—C10	1.381 (2)
C1—C2	1.343 (2)	C9—H9	0.9500
C1—H1	0.9500	C10—C11	1.3824 (19)
C2—C3	1.421 (2)	C10—H10	0.9500
C2—H2	0.9500	C11—H11	0.9500
			
C1—O1—C4	105.83 (11)	O2—C5—C4	121.20 (13)
C5—N1—C6	128.99 (12)	N1—C5—C4	113.25 (12)
C5—N1—H1N	115.0 (12)	N1—C6—C11	121.93 (13)
C6—N1—H1N	115.6 (12)	N1—C6—C7	121.29 (12)
O4—N2—O3	121.93 (12)	C11—C6—C7	116.78 (13)
O4—N2—C7	118.51 (12)	C8—C7—C6	121.60 (13)
O3—N2—C7	119.56 (11)	C8—C7—N2	116.10 (12)
C2—C1—O1	110.73 (13)	C6—C7—N2	122.29 (12)
C2—C1—H1	124.6	C9—C8—C7	120.11 (13)
O1—C1—H1	124.6	C9—C8—H8	119.9
C1—C2—C3	106.62 (13)	C7—C8—H8	119.9
C1—C2—H2	126.7	C8—C9—C10	119.05 (13)
C3—C2—H2	126.7	C8—C9—H9	120.5
C4—C3—C2	106.38 (13)	C10—C9—H9	120.5
C4—C3—H3	126.8	C9—C10—C11	121.58 (14)
C2—C3—H3	126.8	C9—C10—H10	119.2
C3—C4—O1	110.43 (12)	C11—C10—H10	119.2
C3—C4—C5	131.11 (13)	C10—C11—C6	120.86 (13)
O1—C4—C5	118.38 (12)	C10—C11—H11	119.6
O2—C5—N1	125.55 (13)	C6—C11—H11	119.6
			
C4—O1—C1—C2	−0.32 (15)	N1—C6—C7—C8	−179.94 (12)
O1—C1—C2—C3	0.33 (17)	C11—C6—C7—C8	0.84 (19)
C1—C2—C3—C4	−0.21 (16)	N1—C6—C7—N2	−0.61 (19)
C2—C3—C4—O1	0.01 (15)	C11—C6—C7—N2	−179.83 (11)
C2—C3—C4—C5	−176.78 (14)	O4—N2—C7—C8	−10.06 (18)
C1—O1—C4—C3	0.19 (15)	O3—N2—C7—C8	169.34 (12)
C1—O1—C4—C5	177.44 (11)	O4—N2—C7—C6	170.57 (12)
C6—N1—C5—O2	2.7 (2)	O3—N2—C7—C6	−10.03 (19)
C6—N1—C5—C4	−176.32 (12)	C6—C7—C8—C9	−0.4 (2)
C3—C4—C5—O2	−7.1 (2)	N2—C7—C8—C9	−179.82 (12)
O1—C4—C5—O2	176.34 (12)	C7—C8—C9—C10	−0.4 (2)
C3—C4—C5—N1	172.03 (14)	C8—C9—C10—C11	0.8 (2)
O1—C4—C5—N1	−4.55 (17)	C9—C10—C11—C6	−0.3 (2)
C5—N1—C6—C11	−4.7 (2)	N1—C6—C11—C10	−179.66 (12)
C5—N1—C6—C7	176.12 (12)	C7—C6—C11—C10	−0.44 (19)

### Hydrogen-bond geometry (Å, º)

**Table:**

*D*—H···*A*	*D*—H	H···*A*	*D*···*A*	*D*—H···*A*
C2—H2···O2^i^	0.95	2.55	3.3857 (18)	146

Symmetry code: (i) −*x*+1, *y*+1/2, −*z*+3/2.
